# Genetic and inflammatory factors underlying gestational diabetes mellitus: a review

**DOI:** 10.3389/fendo.2024.1399694

**Published:** 2024-04-17

**Authors:** Gyan Watson Ray, Qiaoli Zeng, Phidelia Kusi, Hengli Zhang, Taotao Shao, Taili Yang, Yue Wei, Mianqin Li, Xiaoqun Che, Runmin Guo

**Affiliations:** ^1^ Department of Internal Medicine, Shunde Women and Children’s Hospital (Maternity and Child Healthcare Hospital of Shunde Foshan), Guangdong Medical University, Foshan, China; ^2^ Key Laboratory of Research in Maternal and Child Medicine and Birth Defects, Guangdong Medical University, Foshan, China; ^3^ Matenal and Child Research Institute, Shunde Women and Children’s Hospital (Maternity and Child Healthcare Hospital of Shunde Foshan), Guangdong Medical University, Foshan, China; ^4^ University of Ghana, Ministry of Fisheries and Aquaculture Development, Fisheries Commission, Accra, Ghana; ^5^ Department of Obstetric, Shunde Women and Children’s Hospital (Maternity and Child Healthcare Hospital of Shunde Foshan), Guangdong Medical University, Foshan, Guangdong, China; ^6^ Reproductive Medicine Center, Shunde Women and Children’s Hospital (Maternity and Child Healthcare Hospital of Shunde Foshan), Guangdong Medical University, Foshan, Guangdong, China

**Keywords:** beta-cell dysfunction, genetic factors, gestational diabetes mellitus (GDM), genetic variations, glucose metabolism, inflammatory pathways

## Abstract

Gestational diabetes mellitus (GDM) poses a significant global health concern, impacting both maternal and fetal well-being. Early detection and treatment are imperative to mitigate adverse outcomes during pregnancy. This review delves into the pivotal role of insulin function and the influence of genetic variants, including SLC30A8, CDKAL1, TCF7L2, IRS1, and GCK, in GDM development. These genetic variations affect beta-cell function and insulin activity in crucial tissues, such as muscle, disrupting glucose regulation during pregnancy. We propose a hypothesis that this variation may disrupt zinc transport, consequently impairing insulin production and secretion, thereby contributing to GDM onset. Furthermore, we discussed the involvement of inflammatory pathways, such as TNF-alpha and IL-6, in predisposing individuals to GDM. Genetic modulation of these pathways may exacerbate glucose metabolism dysregulation observed in GDM patients. We also discussed how GDM affects cardiovascular disease (CVD) through a direct correlation between pregnancy and cardiometabolic function, increasing atherosclerosis, decreased vascular function, dyslipidemia, and hypertension in women with GDM history. However, further research is imperative to unravel the intricate interplay between inflammatory pathways, genetics, and GDM. This understanding is pivotal for devising targeted gene therapies and pharmacological interventions to rectify genetic variations in SLC30A8, CDKAL1, TCF7L2, IRS1, GCK, and other pertinent genes. Ultimately, this review offers insights into the pathophysiological mechanisms of GDM, providing a foundation for developing strategies to mitigate its impact.

## 1 Introduction

Gestational diabetes mellitus (GDM) presents a formidable challenge in maternal healthcare, affecting millions of pregnancies worldwide annually. This metabolic disorder, characterized by elevated glucose levels during pregnancy, poses significant risks to both maternal and fetal health (1–4). Recent epidemiological data suggests a staggering impact, with over 21.1 million live births affected globally in 2021 alone, underscoring its prevalence and urgency for attention (2, 5, 6).

Furthermore, emerging scientific insights indicate a substantial rise in GDM incidence, particularly in regions such as Asia, Africa, Europe, and Latin America. Notably, South and Southeast Asia bear the brunt, with over 90% of cases occurring in these regions, followed by the Middle East and North Africa region with 11.7% (2). Interestingly, South American countries also report a high prevalence of GDM, with approximately 15% of pregnant women in Chile and Peru being diagnosed with the disorder over the past two decades (7–9). Contrastingly, advanced economies exhibit comparatively lower prevalence rates, with countries like Australia, Canada, the United States, and the United Kingdom reporting less than 6% of pregnancies affected (10).

The multifactorial nature of GDM is increasingly evident, with genetic predispositions playing a pivotal role in GDM susceptibility, with links to type 2 diabetes mellitus (T2DM) becoming increasingly evident (11). A comprehensive analysis of potential genes associated with GDM has revealed the involvement of crucial genetic markers in GDM susceptibility. Notably, genes such as SLC30A8, CDKAL1, TCF7L2, IRS1, and GCK exhibit polymorphisms that are strongly linked to an increased risk of GDM (11–13). Furthermore, it is imperative to consider histone alterations and other epigenetic changes, including DNA methylation, as they are pivotal in regulating gene expression throughout pregnancy. These mechanisms are essential for maintaining the delicate balance for a healthy gestational period. The implications of these genetic mutations extend beyond the mother’s health, potentially posing risks to the well-being of the fetus as well. Consequently, it becomes crucial to acknowledge the potential impact of these mutations on the offspring’s overall health.

During pregnancy, women with GMD may experience heightened inflammation in their bodies. This inflammatory response is triggered by the increased levels of glucose in the blood characteristic of GDM, leading to elevated levels of cytokines and other inflammatory markers (14). Inflammation plays a crucial role in the pathogenesis of GDM, as it can impair insulin sensitivity and lead to complications such as pre-eclampsia and preterm birth (14). Managing inflammation in pregnant women with GDM is essential for optimizing their outcomes and preventing adverse maternal and fetal health effects. It is crucial to acknowledge the various dimensions of the relationship between genetics, inflammatory pathways, and GDM. Alongside genetic factors, lifestyle choices and other non-genetic variables play a significant role in determining the overall risk and progression of GDM. A more comprehensive understanding of GDM can be gained by examining how genetics influence inflammatory pathways within the context of the condition.

However, despite advancements, gaps persist in our understanding of the precise genetic and inflammatory pathways underpinnings of GDM, warranting further exploration (15). This review seeks to elucidate the roles of key genes, including SLC30A8, CDKAL1, TCF7L2, IRS1, and GCK, in GDM etiology. Delving into molecular inflammatory pathways and genetics aims to provide comprehensive insights that pave the way for tailored interventions and improved maternal-fetal outcomes. It also discusses the SLC30A8 expression and mechanism in regulating placental tissue.

## 2 Genetic basis of gestational diabetes mellitus

Genetic and environmental factors are pivotal in the etiology of gestational diabetes mellitus (GDM), a multifaceted disorder affecting pregnancy. Research indicates a genetic underpinning influenced by various factors, including SLC30A8, CDKAL1, TCF7L2, IRS1, and GCK (16, 17). The intricate relationship between genetics and GDM onset is striking, with a plethora of genes identified as contributors, underscoring the complexity of disease progression (18). Notably, susceptibility to GDM has been linked to genetic variations impacting insulin sensitivity, exemplified by SLC30A8, CDKAL1, TCF7L2, IRS1, and GCK. Ongoing research continues to probe the genetic landscape of GDM, exploring the significant impacts of these genetic factors on pregnancy-associated glucose dysregulation (17). Such insights offer a valuable understanding of GDM’s pathogenesis and avenues for targeted interventions to mitigate its risks.

## 3 Genetic markers and risk prediction

Understanding genetic markers in predicting the risk of developing Gestational Diabetes Mellitus (GDM) has made significant advancements in genetics in recent years. Moreover, during postpartum follow-up, the incidence of type 2 diabetes (T2DM) in women with GDM can range from 50% to 70%. According to (19), GDM in middle-aged women is a major cause of type 2 diabetes. Specific differences in DNA sequences, known as genetic markers, have been identified as potential predictors of GDM risk. Numerous studies have explored the relationship between genetic markers and the likelihood of developing GDM, shedding light on the underlying genetic factors contributing to this condition (19). (20, 21) have conducted research in this area. For instance, these studies have discovered that specific gene variants, such as TCF7L2 and KCNJ11, are associated with a higher risk of GDM (20–22). These results pave the way for the development of personalized risk prediction models and provide valuable insights into the genetic basis of GDM. Incorporating genetic markers into risk prediction models holds excellent potential for early detection and treatment of GDM cases. By integrating genetic data with conventional risk factors like maternal age, body mass index, and family history of diabetes (41), clinicians can enhance the accuracy of risk assessment. This approach enables the implementation of targeted interventions, such as dietary modifications and careful monitoring, to mitigate the risks associated with GDM.

## 4 Diagnosis of gestational diabetes mellitus

The importance of early detection of gestational diabetes mellitus (GDM) cannot be overstated, as it allows for timely intervention and management strategies. According to (23), the prevalence of GDM among pregnant women can range from 15% to 70%, underlining the critical need for robust screening protocols. Early identification of GDM empowers medical providers to implement tailored interventions, such as dietary adjustments, exercise regimens, and, when necessary, insulin therapy (23). A study conducted by (24) found that early diagnosis and management of GDM significantly reduced the risk of adverse perinatal outcomes, such as macrosomia and neonatal hypoglycemia. By mitigating risks such as pre-eclampsia, macrosomia, and neonatal hypoglycemia, early diagnosis serves as a protective shield for both maternal and fetal health. The ramifications of accurate diagnosis extend beyond immediate management; they pave the way for optimal outcomes throughout the pregnancy journey.

In the field of GDM diagnosis, two prominent criteria endorsed by reputable bodies stand out: the International Association of Diabetes and Pregnancy Study Groups (IADPSG) and the American Diabetes Association (ADA) criteria (25, 26). Both rely on oral glucose tolerance tests (OGTT) to assess glucose metabolism during pregnancy, but they offer distinct approaches to diagnosis. The IADPSG criteria advocate a streamlined, one-step approach, with a 75g OGTT at 24-28 weeks of gestation. A fasting plasma glucose level of ≥92 mg/dL (5.1 mmol/L) or a 2-hour plasma glucose level of ≥180 mg/dL (10.0 mmol/L) signals GDM (25). Notably, studies by (27, 28) have validated the effectiveness of these criteria, particularly in Asian populations like China, underscoring their global relevance. Furthermore, the International Federation of Gynecology and Obstetrics (FIGO) endorsed the use of the International Association of Diabetes and Pregnancy Study Groups (IADPSG) criteria due to its global applicability and ability to identify women at risk more accurately (29).

On the other hand, the ADA criteria, predominant in the United States, favor a two-step approach. This involves a 50g glucose challenge test (GCT) followed by OGTT if the former yields elevated glucose levels (30). However, discrepancies in diagnostic thresholds and approaches among healthcare professionals underscore the need for standardization. In response, the ADA has proposed adjustments, advocating for a lower fasting plasma glucose level as the primary diagnostic test (31). Furthermore, emerging evidence suggests seasonal variations in GDM prevalence, emphasizing the multifactorial nature of its diagnosis (32). GDM’s complex landscape demands a unified approach to diagnosis and management. The IADPSG criteria offer a comprehensive, global framework, while the ADA criteria cater specifically to the U.S. context. Yet, harmonizing these approaches and implementing standardized diagnostic protocols remain imperative.

**Figure 1:**
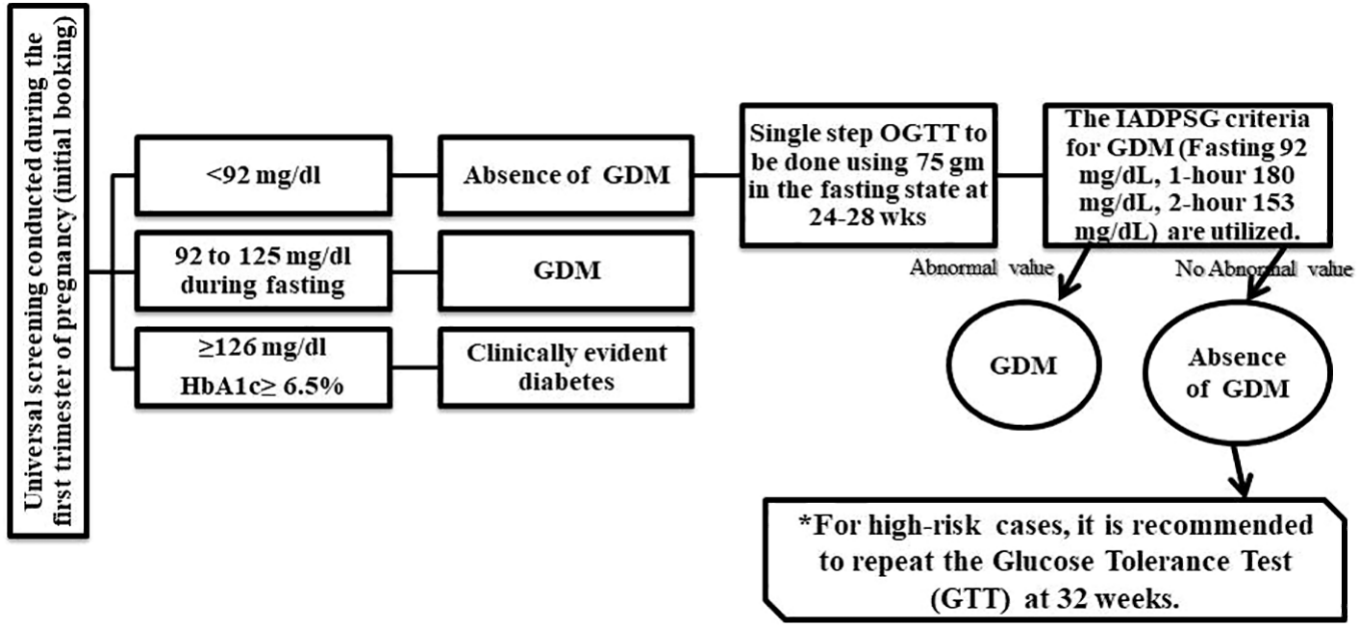
General diagnostics process of GDM identification. Source.

Accurate and timely diagnosis of GDM is pivotal for ensuring the well-being of both mother and child. By embracing evidence-based recommendations and fostering collaboration among healthcare providers, we can refine diagnostic practices, enhance patient care, and ultimately, safeguard the health outcomes of pregnant individuals and their offspring. The proposed diagnostic process in **Figure 1** serves as a visual reminder of the journey toward effective GDM identification and management on a global scale.

### 4.1 Guidelines for screening the gestational diabetes mellitus during early pregnancy

It is widely recommended that women who have a high risk of pre-existing diabetes undergo screening for this condition during the first trimester of their pregnancy or at the onset of antenatal treatment (33). To better understand the populations that should be screened, **Table 1** offers a comprehensive overview based on the recommendations of national guideline organizations. Recent research studies have identified various genes that are associated with gestational diabetes mellitus (GDM). These genes play a crucial role in the development and progression of GDM during pregnancy. The **Table 2** below provides a list of these genes and their significance in GDM research.

**Table 1:** Guidelines for Screening the Gestational Diabetes Mellitus during Early Pregnancy.

Organization	Recommended Populace	References
World Health Organisation (WHO)	Depending on available resources, conflicting priorities, and the prevalence of glucose intolerance within the local population, it is imperative for each country or health service to make a well-informed decision regarding the patients who should undergo screening.	(33)
American College of Obstetricians and Gynaecologists (ACOG)	Women who are overweight or obese (with a BMI of ≥25 kg/m^2^ or ≥23 kg/m^2^ in Asian Americans) and have one or more additional risk factors should be identified for further evaluation. These risk factors include physical inactivity, belonging to a high-risk race or ethnicity, having a history of delivering a baby weighing ≥4 kg, having a history of gestational diabetes mellitus, having hypertension (with a blood pressure reading of 140/90 mmHg or taking antihypertensive therapy), having low levels of high-density lipoprotein (HDL) cholesterol (<35 mg/dl or 0.9 mmol/l) and triglycerides, having polycystic ovary syndrome (PCOS), having a hemoglobin A1c (HbA1c) level of ≥5.7%, having impaired glucose tolerance or impaired fasting glucose on previous testing, cardiovascular disease.	(34)
International Association of Diabetes and Pregnancy Study Groups (IADPSG)	The decision is made considering the prevalence of impaired glucose tolerance in the local community, as well as the individual circumstances of each woman, particularly those who are at a higher risk	(35)
Diabetes Canada	Identification of Women at High Risk for Undetected Type 2 Diabetes (T2D)	(36)
American Diabetes Association (ADA)	Women who have one or more risk factors for diabetes mellitus (DM) should be aware of the following: belonging to a high-risk race or background, experiencing hypertension (with a blood pressure reading of 140/90 mmHg or taking antihypertensive therapy), having a first-degree family with DM, being diagnosed with polycystic ovarian syndrome (PCOS), having a history of cardiovascular disease, women who have previously experienced gestational diabetes mellitus (GDM) should undergo regular screening for DM, at least once every three years. having low levels of high-density lipoprotein (HDL) cholesterol (<35 mg/dl or 0.9 mmol/l) and increased levels of triglycerides, leading a physically inactive lifestyle,	(37)

**Table 2:** Various genes associated with gestational diabetes mellitus (GDM) in recent research studies.

Genes	Sample size	Gene function	References
CDKAL1; CDK5 regulatory subunit associated protein 1 like 1	GDM n = 10336; Control n = 17,445	The gene CDKAL1 encodes a member of the methylthiotransferase family. While the exact function of CDKAL1 remains unclear, it has been associated with both risk variants that are linked to decreased insulin secretory capacity and glucose-stimulated insulin secretion [51]. This gene locus has also been linked to type 2 diabetes (T2D) Cho, Y. M., 2009; Ohara-Imaizumi, Mica, 2010	(38)
GCK; glucokinase	29 studies	The gene encodes the hexokinase GCK, which plays a crucial role in the pancreas’s response to glucose. It is responsible for the secretion of insulin and also influences the liver’s absorption of glucose and its conversion into glycogen. This gene locus has been linked to Type 2 Diabetes (T2D).	(17)
TCF7L2; transcription factor 7-like 2	GDM patients = 5485; Healthy control = 347,856	The gene encodes a transcription factor that contains a specific box, which plays a crucial role in the Wnt signaling pathway. This transcription factor belongs to the high mobility group and is also involved in the synthesis and processing of insulin Zhou, Yuedan, 2014. Furthermore, this gene locus is linked to Type 2 Diabetes (T2D).	(39)
CDKAL1	GDM patients 835 and Healthy Control 870	Beta-cell dysfunction and reduced insulin secretion	(12)
IRS1	213 GDM patients including fetuses and 191 Healthy control including fetuses	Insulin-stimulated signaling pathways regulate insulin receptor substrate (IRS) activity in various tissues, including muscle and pancreatic beta cells, and have been identified as a pathogenic factor in diabetes onset (Yiannakouris, 202).	(40)
SLC30A8	500 patients with GDM and 502 control subjects.	The SLC30A8 gene, encoding a crucial zinc transporter, may be affected by mutations that affect pancreatic beta cell function, potentially increasing gestational diabetes mellitus susceptibility.	(41)

Gestational Diabetes Mellitus= GDM; Sample size= n.

## 5 The role of genetic factors in the development of gestational diabetes

Gestational Diabetes Mellitus (GDM) is a critical condition during pregnancy characterized by specific glucose level thresholds outlined by the (31). These thresholds, including fasting glucose levels, oral glucose tolerance test (OGTT) results, and symptomatic hyperglycemia, serve as crucial indicators for diagnosing and managing GDM. The research underscores the profound impact of GDM on maternal and fetal health. The excessive presence of glucose, fatty acids, and amino acids in maternal circulation can lead to the development of larger-than-average fetuses, posing risks during delivery and increasing the likelihood of cesarean sections (42). Moreover, the metabolic dysregulation associated with GDM, marked by hyperglycemia and insulin resistance, can instigate a cascade of adverse effects (42). This metabolic upheaval not only heightens the oxygen demand but also induces chronic hypoxia and inflammation, contributing to further complications.

Moreover, the repercussions of GDM extend far beyond pregnancy. Both mothers who experience GDM and their offspring face an elevated risk of developing type 2 diabetes, obesity, and cardiovascular conditions later in life (42). While genetic factors undoubtedly play a role, it’s crucial to recognize the multifaceted nature of GDM’s etiology. Recent studies have delved into the intricate interplay between genetics, epigenetics, environmental factors, and the body’s microbiota in predisposing individuals to GDM (43, 44). (42) demonstrated that the surplus of glucose, fatty acids, and amino acids characteristic of GDM contributes to fetal macrosomia, chronic hypoxia, and inflammation, underscoring the multifactorial nature of its pathogenesis. Moreover, investigation by (10) has highlighted the role of epigenetic mechanisms and environmental factors in predisposing individuals to GDM, further reinforcing the complexity of its etiology.

The emergence of epigenetic signatures and their association with GDM highlights the need for a comprehensive understanding of the condition. Moreover, investigations into common genetic variants linked to type 2 diabetes, such as TCF7L2, SLC30A8, and CDKAL1, underscore the interconnectedness of GDM and metabolic disorders (45). A striking finding from these studies is the overlap of specific genetic variants associated with diabetes in non-pregnant populations with those prevalent in women with GDM (46–48). Variants in genes like CDKN2A-CDKNA2B, TCF7L2, KCNQ1, MTNR1B, and FTO have been consistently observed in women with GDM across diverse populations (46–49). Emerging research focuses on genes associated with β-cell function and insulin secretion, with TCF7L2, SLC30A8, and GCK among the implicated candidates (45). These findings underscore the importance of understanding the molecular mechanisms underlying GDM for targeted intervention and management strategies. This convergence reinforces the notion that GDM shares genetic underpinnings with type 2 diabetes, emphasizing the importance of targeted interventions and preventive measures.

### 5.1 Role of SLC30A8 in the development of GDM

SLC30A8, also known as zinc transporter 8 (ZnT8), is a pivotal gene implicated in the pathogenesis of Gestational Diabetes Mellitus (GDM) (50). This gene regulates zinc homeostasis by facilitating zinc transport into pancreatic beta cells, which is crucial for insulin synthesis and secretion (51). Several studies have indicated that variations in the SLC30A8 gene influence susceptibility to GDM (52–54). Variations in SLC30A8 have been associated with an increased risk of GDM development. Specific single nucleotide polymorphisms (SNPs) within the gene alter protein structure and function, impairing zinc transportation and, subsequently insulin secretion (55, 56). Consequently, disrupted insulin production leads to elevated glucose levels during gestation, contributing to GDM diagnosis.

The research underscores the critical role of SLC30A8 in maintaining beta cell function and mass (55). Beta cells rely heavily on zinc as a cofactor for insulin synthesis (57, 58). Dysfunctional SLC30A8 results in zinc depletion within beta cells, causing impaired function and decreased survival. This dysfunction further disrupts glucose regulation, exacerbating GDM susceptibility. ZnT8, the protein encoded by SLC30A8, facilitates the movement of zinc ions from the cytoplasm to insulin granules within pancreatic beta cells. Insulin granules contain high zinc concentrations, and the co-secretion of zinc with insulin impacts neighboring endocrine cells (59, 60). Studies reveal the complex role of zinc in glucose-stimulated insulin secretion (GSIS), with ZnT8 influencing this process significantly (61, 62).

While some studies demonstrate the stimulatory effect of zinc on insulin secretion, others suggest an inhibitory role (59, 63). The intricate relationship between zinc and insulin secretion remains a subject of ongoing research. Nonetheless, the critical involvement of SLC30A8 and ZnT8 in insulin secretion underscores their potential influence on GDM susceptibility. Additionally, a study in animal models highlights the potential therapeutic benefits of zinc supplementation in restoring beta cell function and mitigating GDM risk (51). Intraperitoneal zinc supplementation in rats restored ZnT8 levels and improved beta cell function, emphasizing the therapeutic potential of targeting SLC30A8 in GDM management (51).

#### 5.1.1 Link between altered SLC30A8 expression in placental tissue and gestational diabetes mellitus

The SLC30A8 gene on chromosome 8q24.11 has garnered significant attention in the study of type 2 diabetes (T2D). Recent research has highlighted its relevance to GDM, as certain variations in the SLC30A8 gene may impact a mother’s ability to metabolize glucose during pregnancy (41). The placenta, a vital organ for fetal development, undergoes dynamic changes in gene expression throughout pregnancy. It serves as a gateway, controlling the flow of nutrients between the mother and the developing fetus (64). Understanding the intricate mechanisms involved in placental function is crucial for comprehending the complexities of fetal development and maternal health.

Placental zinc transporters play a vital role in maintaining the delicate balance of zinc levels in the developing fetus. These transporters are regulated as part of complex processes that are not yet fully understood. Zinc, a crucial element, serves as a catalyst or structural component in hundreds of proteins, making it essential for various physiological functions. Insufficient zinc levels can lead to a range of symptoms, including skin lesions, compromised immune function, growth retardation, and GDM in pregnant women (65). Research has shown that the amount of zinc influences the expression of zinc transporters in mouse placentas in their diet (66, 67). However, even with moderate dietary zinc restriction, this modulation of expression is insufficient to support optimal fetal nutrition. The SLC30A8 gene encodes ZnT8, a protein that regulates zinc transport across cellular membranes. Zinc is crucial for healthy trophoblast differentiation, fetal growth, and protection against oxidative stress in the placenta. Altered expression of SLC30A8 can disrupt these processes, contributing to the development of GDM (66).

Research findings indicate that the expression of SLC30A8 varies in the placental tissue of patients with GDM compared to pregnancies with normal blood sugar levels (66, 68). This variation in SLC30A8 expression suggests a potential disruption in zinc homeostasis and its impact on fetal development and metabolic programming. Moreover, a decrease in insulin function leads to an upregulation of genes associated with placental fatty acid β-oxidation and transport (69). This upregulation results in a higher transfer of long-chain polyunsaturated fatty acids (LCPUFA) to the developing fetus. However, elevated lipid levels directed toward the fetus have the potential to cause obesity and metabolic disturbances later in life (69).

Abnormalities in placental function have been closely associated with unfavorable pregnancy outcomes such as macrosomia, premature birth, and neonatal problems. Therefore, examining the relationship between modified SLC30A8 expression and GDM provides a novel insight into the potential effects of placental disruptions on fetal development and metabolic programming. As research continues to unravel the intricate molecular landscape of GDM, the link between altered SLC30A8 expression in placental tissue and the development of GDM emerges as a promising area of investigation. Further studies are warranted to elucidate the precise mechanisms underlying this association and to explore therapeutic intervention interventions that could positively influence pregnancy outcomes in women at risk for GDM.

### 5.2 Role of insulin receptor substrate gene in the development of GDM

Insulin resistance (IR) serves as the fundamental pathogenesis of Gestational Diabetes Mellitus (GDM), initiating its onset (70). While the precise mechanism remains elusive, inflammation is a critical contributor to insulin resistance and pancreatic beta-cell dysfunction. Studies have highlighted the significance of the IRS1 gene in insulin signaling, with genetic variations linked to insulin resistance, a pivotal factor in GDM development (71, 72). The IRS1 gene encodes the IRS1 protein, crucial for transmitting insulin signals and regulating glucose metabolism (73, 74). However, mutations or variations in IRS1 can disrupt these signaling pathways, leading to impaired insulin action (73).

Furthermore, IRS1 governs insulin activity in various tissues, including muscle, adipose tissue, and pancreatic beta cells, underscoring its role in glucose regulation (75). Notably, the IRS1 rs2943641 polymorphism, located downstream of the IRS1 gene, has been associated with increased fasting hyperinsulinemia and reduced insulin sensitivity (76, 77). This polymorphism has also been linked to elevated risk of type 2 diabetes and higher fasting glucose levels in women with GDM (78). Additionally, research suggests a correlation between IRS1 gene expression in the placenta of pregnant women with GDM and increased body mass index, further highlighting its significance in GDM pathogenesis (79).

Studies in mice have shown that IRS1 or IRS2 signals regulate hepatic gene expression, which is crucial for glucose homeostasis and systemic growth (80). Therefore, pregnant women with genetic variations in the IRS1 gene may struggle to maintain optimal sugar levels during pregnancy, heightening their risk of GDM (80). Alongside IRS1, the SLC30A8 gene also significantly influences GDM development through its role in zinc homeostasis regulation and insulin and beta cell function (41). Variations in both SLC30A8 and IRS1 genes can impair zinc transportation and subsequent insulin secretion in beta cells, increasing GDM risk. Studying the mechanisms underlying SLC30A8’s influence on GDM development holds promise for early detection, targeted intervention, and personalized treatment strategies for pregnant women at higher risk of GDM. However, the genetic bases of GDM remain incompletely understood, urging future studies to identify various genetic markers, mechanism genes, and environmental factors contributing to GDM development.

### 5.3 Role of TCF7L2 in the development of GDM

TCF7L2 is an extensively studied genetic factor implicated in reduced insulin secretion and an increased risk of developing gestational diabetes mellitus (GDM). It was the first locus firmly identified through genomic linkage studies and is considered the most influential locus for the risk of type 2 diabetes (T2D) (11). As one of the key transcription factors in the Wnt signaling pathway, TCF7L2’s functional domains closely relate to highly conserved sequence regions within the gene. Its consistent replication across diverse populations with various genetic backgrounds underscores its significance as one of the strongest genetic associations with complex diseases found in research studies.

A meta-analysis of genetic associations observed in different populations revealed that TCF7L2 variants linked with type 2 diabetes operate through a multiplicative genetic model. It was estimated that TCF7L2 contributes to nearly 20% of T2D cases (81). However, the specific mechanisms through which TCF7L2 influences GDM and T2D remain not fully understood (11). Therefore, further research is necessary in this field to gain a deeper understanding of the role played by TCF7L2 in the development of GDM.

### 5.4 Role of glucose kinase in the development of GDM

One crucial enzyme involved in regulating glucose uptake and storage is glucose kinase (GCK) (82). Additionally, GCKR, or glucokinase regulatory protein, has been identified as the rate-limiting factor for GCK, as highlighted by (83). GCK and GCKR work in tandem to maintain glucose homeostasis. Notably, these genes have been linked to an increased susceptibility to type 2 diabetes through genome-wide association studies (GWAS) (84). Significant differences were observed in age, pre-gestational BMI, education level, and family history of diabetes when comparing the case and control groups (P < 0.05) (85). Despite accounting for these confounding factors, GCK rs1799884 remained significantly associated with gestational diabetes mellitus (GDM) (P < 0.05). However, no significant associations were found between GDM odds and the polymorphisms rs4607517, rs10278336, rs2268574, rs780094, and rs1260326 (P > 0.05) (85).

Furthermore (85), discovered that pregnant women with the rs4607517 TT genotype exhibited significantly higher fasting glucose levels compared to those with the CC genotype (P < 0.05). The Chinese population, in particular, faces an elevated risk of developing GDM due to the GCK rs1799884 mutation. Further extensive research is warranted to understand better the relationship between GCK and GCKR polymorphisms and susceptibility to GDM.

### 5.5 Role of CDKAL1 in the development of GDM

Beta-cell dysfunction and reduced insulin secretion have been found to be associated with CDKAL1, as stated by (86). These genetic variations can lead to disruptions in glucose homeostasis during pregnancy, potentially resulting in elevated sugar levels. The CDKAL1 gene encodes Cyclin-dependent kinase 5 regulatory subunit-associated protein 1 (CDK5RAP1)-like 1. CDK5, a serine/threonine protein kinase, plays a critical role in the pathophysiology of β-cell dysfunction and the predisposition to type 2 diabetes (T2DM) by regulating insulin secretion in a glucose-dependent manner, as highlighted by (87). However, further studies are necessary to comprehend the complete genetic landscape fully. Future research is crucial in enhancing risk prediction and developing more effective preventive strategies and personalized care for pregnant individuals at risk for gestational diabetes mellitus (GDM).

**Figure 2:**
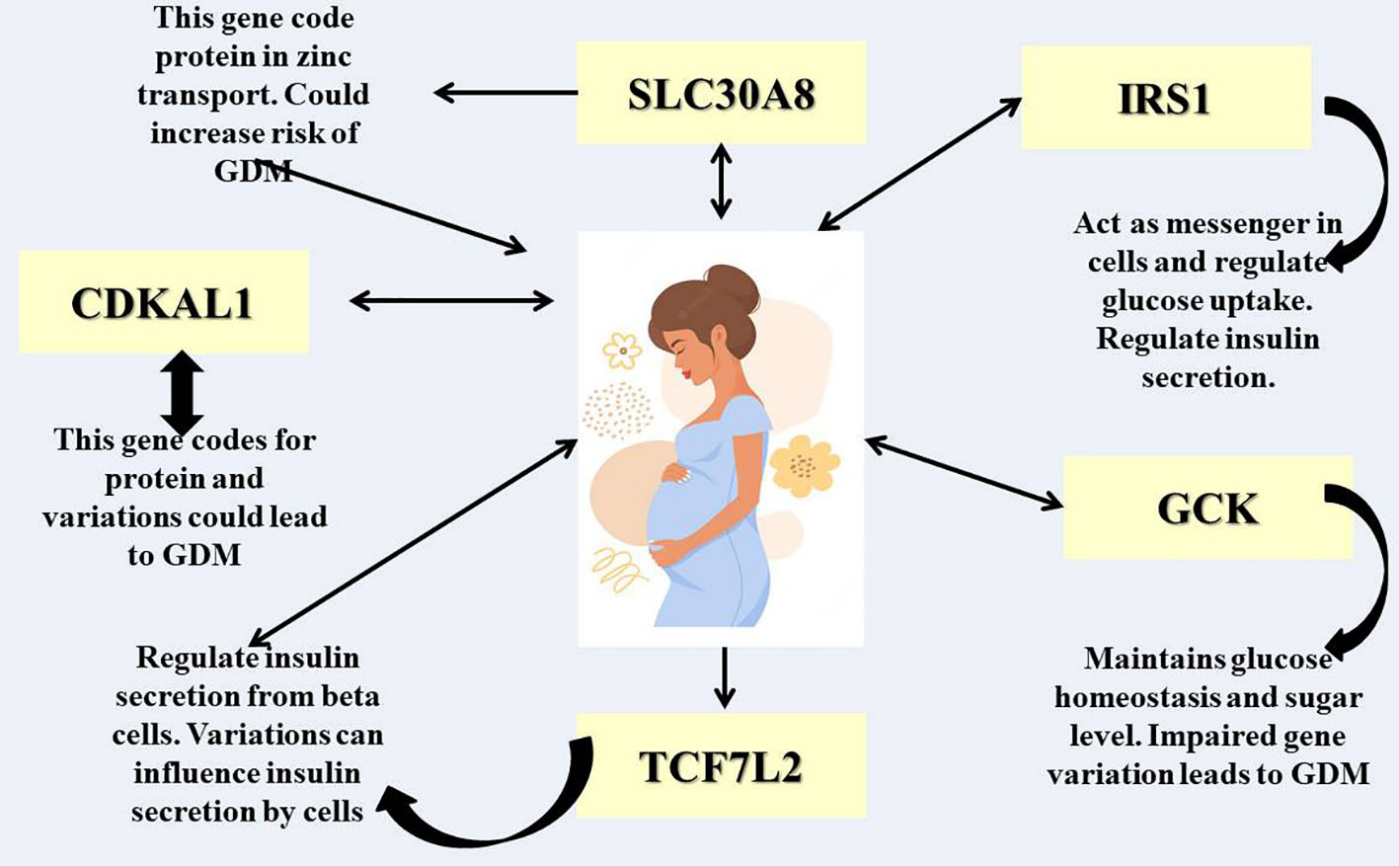
Different genetic factors responsible for GDM.

**Figure 2** illustrates a visual representation of various genes that could potentially be linked to gestational diabetes mellitus (GDM), a condition characterized by glucose intolerance during pregnancy. The diagram features a central image of a pregnant woman, with multiple gene acronyms connected by arrows indicating potential interactions, functions, and regulatory pathways. These genes may influence an individual’s susceptibility to or progression of GDM, highlighting the significant role of heredity in the development of this disease.

## 6 Impacts of environmental factors and the lifestyle modification that could impact GDM

Epigenetic research has demonstrated that pregnancies affected by GDM exhibit distinct gene methylation statuses compared to those without GDM (88). This growing body of evidence indicates that GDM not only affects the duration of pregnancy but also impacts the development of the offspring. Consequently, these effects can lead to long-term consequences and unfavorable health outcomes for the offspring. Epigenetic modifications can occur through three mechanisms: histone modification, DNA methylation, and impaired function of non-coding ribonucleic acids (ncRNAs), including microRNAs (miRNAs) (89). These findings shed light on the complex interplay between epigenetic factors and the development of GDM.

In addition, environmental factors and the lifestyle of the mother can give rise to epigenetic modifications that influence the likelihood of the offspring developing GDM. Stress, inadequate diet, and exposure to environmental pollutants during pregnancy can all induce epigenetic alterations that impact the expression of genes involved in glucose metabolism. These genetic and epigenetic factors may affect the functioning of beta cells and insulin resistance, thereby playing a role in the development of GDM. In recent years, molecular biomarkers have garnered significant attention in the field of GDM prognosis, diagnosis, and screening. We propose that genetic and epigenetic modifications contribute to the underlying mechanisms of GDM. By exploring the interplay between environmental variables, maternal lifestyle, and epigenetic changes, we can gain a deeper understanding of the pathophysiology of GDM. This knowledge can pave the way for improved prognosis, diagnosis, and screening methods for this condition.

## 7 The role of single nucleotide polymorphisms in gestational diabetes mellitus susceptibility

Single Nucleotide Polymorphisms (SNPs) represent the most prevalent form of genetic variation observed in human DNA (90). These subtle alterations in a single nucleotide base can have profound implications for an individual’s vulnerability to various diseases, including GDM (Gestational Diabetes Mellitus) (90). SNPs are nucleotide variations occurring at single positions in the DNA sequence (91). Thanks to current genotyping technologies such as SNPscan™, TaqMan SNP genotyping, nucleic-acid-modifying enzymes, and PCR, we can now identify disease-related SNPs (41, 52, 92), including those linked to metabolic disorders such as obesity, TDM2 (Type 2 Diabetes Mellitus), cardiovascular disease, and GDM (93, 94). These SNPs may be associated with a higher or lower risk of developing GDM in different populations worldwide (49, 95–99).

Numerous studies have identified specific SNPs significantly associated with GDM susceptibility (100–102). These genetic variants are often found in genes involved in insulin secretion, insulin resistance, and glucose metabolism pathways. A study conducted by (100, 102–104) revealed that SNPs associated with T2DM also confer a significant risk for GDM in a multi-ethnic cohort comprising individuals from Hawaii, Korea, and China. In these studies, the researchers gathered information from 291 women with GDM diagnoses and 734 matched non-diabetic controls (197 non-diabetic controls; Filipinos: 162 GDM, Japanese: 58 GDM, Pacific Islanders: 71 GDM, 395 controls; 142 controls). Twenty-five different SNPs linked to 18 different loci were genotyped, and their allele frequencies were calculated using maternal DNA (100, 103, 104).

After adjusting for various factors such as age, body mass index (BMI), parity, and gravidity through multivariable logistic regression, the researchers identified several SNPs that exhibited significant associations with GDM, with these associations being specific to different ethnicities (103, 105). Additionally, substantial correlations with GDM were found among Filipinos for the SNPs rs2237895 (KCNQ1), rs1113132 (EXT2), rs2237892 (KCNQ1), rs1111875 (HHEX), rs10830963 (MTNR1B), and rs13266634 (SLC30A8). SNPs rs4402960 (IGFBP2) and rs2237892 (KCNQ1), on the other hand, were discovered to be strongly related to GDM in Japanese people (100, 103, 104). Finally, SNPs rs10830963 (MTNR1B) and rs13266634 (SLC30A8) showed substantial correlations with GDM among Pacific Islanders (100, 101, 103). These studies highlight the potential of SNPs as biomarkers and deepen our understanding of the molecular mechanisms underlying beta-cell activity and the etiology of GDM for assessment.

## 8 Gene-environment interactions in GDM

Within gestational diabetes mellitus (GDM), the intricate interplay between genetic and environmental factors sparks a thought-provoking discourse. Research indicates that while genetic predispositions may heighten an individual’s susceptibility to GDM, environmental influences often interact with the condition, triggering the manifestation of symptoms (15). The prevalence of GDM has surged, emerging as a significant global health concern with far-reaching implications for public health, particularly in light of the escalating rates of maternal obesity. In addition to immediate adverse effects such as fetal macrosomia and hypertensive pregnancy complications, GDM has been associated with long-term consequences, including heightened cardiometabolic morbidity in both the mother and child (106).

Furthermore, extensive research has unveiled specific genetic variations that are linked to the risk of GDM, offering valuable insights into the hereditary component of the condition (15). It is crucial to comprehend that genes do not always contain all the necessary information. Environmental factors such as maternal stress, physical activity, and nutrition significantly influence the development of GDM. In a study conducted by (107), a gene-lifestyle relationship was discovered between the onset of GDM and the TCF7L2 rs7903146 polymorphism, specifically in relation to adherence to a Mediterranean diet. The study revealed that dietary intervention only alters the risk of developing GDM in individuals carrying the T-risk allele. Women who exhibited moderate to high adherence to the Mediterranean diet demonstrated a lower risk of acquiring GDM than those with low adherence (107).

In a subsequent study conducted by (108), it was observed that women with a history of GDM and a specific MC4R genotype, which increases the risk of developing diabetes and obesity, may derive more incredible benefits from a lifestyle intervention aimed at reducing insulin resistance compared to women with a non-risk genotype. To illustrate, if a woman finds herself in an environment that aligns with her genetic predisposition, she may be able to avoid acquiring GDM. The interplay between genes and lifestyle has been shown to indicate that individuals who are genetically more prone to GDM may experience more tremendous advantages by adhering to a nutritious diet or engaging in physical exercise, as suggested by (15). Conversely, an individual with a lower genetic predisposition could still develop GDM despite unfavorable environmental circumstances. This dynamic exchange underscores the importance of adopting a comprehensive strategy to comprehend and prevent GDM. Personalized therapies could potentially be facilitated through further research into the intricate interaction between genes and the environment. In terms of managing and preventing GDM, tailored interventions based on an individual’s genetic profile and environmental circumstances may yield superior outcomes. However, within the scientific and medical communities, thorough investigation and deliberation are imperative due to ethical concerns and the intricate nature of these associations.

## 9 Mechanisms underlying genetic predisposition

Gestational diabetes mellitus (GDM) is a condition that arises due to a combination of genetic and environmental factors. Extensive research has shown that specific genes related to glucose metabolism, beta-cell function, and insulin resistance play a crucial role in predisposing individuals to GDM (109). Additionally, epigenetic changes occurring during pregnancy, influenced by factors such as maternal nutrition and lifestyle, can further increase the risk of developing GDM. During pregnancy, the body undergoes various adaptations to ensure optimal nutrient delivery to the developing fetus. One key aspect is the regulation of glucose levels. Studies conducted on thin and healthy women using a hyperinsulinemic-euglycemic clamp technique have revealed significant changes in glucose management during the third trimester compared to pre-pregnancy (110, 111). Specifically, basal endogenous glucose production increases by 30%, while insulin sensitivity decreases by 56%. In individuals with normal glucose tolerance, the pancreatic beta cells adapt to these changes by producing higher levels of insulin, which helps maintain normal glucose levels (112). To illustrate, a small study involving normal controls found that the first- and second-phase insulin response to an intravenous glucose tolerance test increased approximately threefold during late pregnancy compared to pre-pregnancy (113–115). Furthermore, postpartum glucose clamp studies have shown that the insulin resistance associated with normal pregnancy resolves within days after delivery, indicating that placental factors play a role in these changes (116).

## 10 Inflammatory pathways in pregnancy

The physiological state of pregnancy is a complex process that necessitates significant changes in the mother’s body, including alterations to the immune system. Throughout pregnancy, the immune system plays a crucial role in maintaining a delicate balance between the fetus’s tolerance and protection from external pathogens. Inflammation is a key component of this immune response, contributing to various processes such as placental development, implantation, and the initiation of labor. Disruption of inflammatory pathways can lead to adverse pregnancy outcomes, underscoring the importance of understanding these mechanisms. The mother’s immune system undergoes substantial changes during pregnancy to establish tolerance to the semi-allogeneic fetus while also preserving the ability to combat infections. These adaptations involve modifications to tissue remodeling processes, cellular responses, and cytokine profiles. Pro-inflammatory cytokines like Interleukin (IL)-6, tumor necrosis factor-alpha (TNF-α), and IL-1β play pivotal roles in initiating and regulating inflammatory responses crucial for implantation, placentation, and the onset of labor (117).

Inflammatory reactions related to pregnancy serve a dual purpose, supporting both the healthy progression of pregnancy and contributing to pathology in cases of dysregulation. Controlled inflammation is vital for the formation of the placenta and embryo implantation early in pregnancy. Immune cells release cytokines and chemokines that facilitate trophoblast cell invasion and the remodeling of the mother’s spiral arteries, both of which are essential for proper placental perfusion (118). During pregnancy, a regulatory environment is established to protect the fetus from an exaggerated response by the mother’s immune system. However, an imbalance in pro- and anti-inflammatory factors can lead to complications such as preterm labor, gestational diabetes, and eclampsia (119). For instance, oxidative stress resulting from excessive inflammation is a key feature of preeclampsia, a condition characterized by high blood pressure and damage to organ systems.

The nuclear factor kappa B (NF-κB) pathway, a transcription factor, regulates the expression of genes involved in immune and inflammatory responses (120). This pathway plays a critical role in immune responses during pregnancy by stimulating the release of pro-inflammatory cytokines, chemokines, and adhesion molecules. Ligands for interleukins I (IL-1) and II (IL-6) play a critical role in fever and the acute phase response as key mediators of inflammatory reactions (121). Elevated levels of IL-6 have been associated with preterm birth and other pregnancy complications (121). Toll-like receptors (TLRs) are responsible for initiating inflammatory responses by recognizing molecular patterns associated with pathogens. The TLR signaling in the placenta is vital for protecting the fetus against infections, but it must be carefully regulated to prevent excessive inflammation (122, 123). The development of therapeutic strategies to modulate these pathways is gaining traction in an effort to improve pregnancy outcomes. For instance, there is ongoing research on therapies targeting TLRs or specific cytokines for the treatment or prevention of pregnancy and cancer complications (124, 125).

Moreover, the placenta serves as a crucial interface for the exchange of nutrients and gases between the mother and the fetus, while also playing a key role in modulating the fetal immune system. It secretes various hormones, growth factors, and cytokines that regulate the mother’s immune system and promote fetal tolerance. The unique cellular composition and structure of the placenta, including trophoblast cells, are essential for its immunomodulatory functions (126).

Furthermore, it is important to recognize that inflammatory pathways during pregnancy can be significantly influenced by various environmental and lifestyle factors. These factors can ultimately affect the health of both the mother and the fetus. For instance, environmental pollutants such as particulate matter and endocrine-disrupting chemicals have the potential to trigger systemic inflammation and disrupt placental function, leading to adverse pregnancy outcomes (127). Similarly, lifestyle choices such as nutrition, exercise, and stress management can also play a crucial role in modifying a mother’s inflammatory responses and influencing the progression of pregnancy. Research has shown that adhering to a well-balanced diet rich in anti-inflammatory nutrients and engaging in regular, moderate exercise can lower the risk of pregnancy complications (128). It is evident that both environmental and lifestyle factors can significantly impact inflammatory pathways during pregnancy, highlighting the importance of maintaining a healthy lifestyle and minimizing exposure to harmful pollutants for the well-being of both the mother and the developing fetus.

## 11 Inflammatory pathways and their genetic regulation in relation to GDM

Inflammation plays a crucial role in the immune system’s defense against infections and tissue damage. However, immune responses can also lead to septic shock, hypersensitivity reactions (such as atopy, anaphylaxis, delayed-type hypersensitivity, and contact hypersensitivity), and the rejection of tissue or organ transplants. Chronic inflammation can occur when immune responses become abnormal or uncontrolled (129). The interplay between inflammatory responses and genetic factors significantly influences the development and progression of gestational diabetes mellitus (GDM).

Research studies have indicated that changes in genes associated with inflammatory pathways may influence an individual’s susceptibility to GDM (130, 131). This research sheds light on a potential cause and target for GDM by establishing a correlation between inflammation and the development of GDM (132). For instance, alterations in inflammatory responses have been found to be associated with variations in genes involved in the production and regulation of cytokines, such as TNF-alpha and IL-6, in pregnant women with GDM (130, 131, 133). Furthermore, it has been observed that the body undergoes a gradual onset of low-grade systemic inflammation following conception (134).

Multiple studies have consistently shown that the development of insulin resistance (IR) is triggered by inflammatory factors (88, 131). The placental tissue possesses robust endocrine functions, capable of producing and releasing a wide array of inflammatory cytokines that exacerbate maternal IR and the chronic inflammatory response. Among these inflammatory factors are TNF-α, IL-6, IL-8, NK-κB, and IL-1β, which are secreted by the placental tissue. IL-1β, as a pro-inflammatory cytokine, induces apoptosis in islet β cells and activates the NK-κB pathway, leading to the release of IL-6, IL-8, and other inflammatory factors (133, 135). IL-6 and IL-8, in turn, stimulate various lymphoid and inflammatory cells, further intensifying the inflammatory response. Additionally, TNF-α is recognized as an independent risk factor for GDM due to its ability to impede insulin signal transmission and glucose transport (133, 135, 136).

The genetic regulation of these pathways may influence the dysregulation of glucose metabolism observed in GDM patients. However, it is crucial to acknowledge that the relationship among inflammatory pathways, genetics, and GDM encompasses multiple dimensions. Apart from genetic factors, lifestyle choices, and other non-genetic determinants play a pivotal role in determining the overall risk and progression of GDM. By delving into the genetic regulation of inflammatory pathways within the context of this disease, a more intricate understanding of GDM emerges.

## 12 Adipokines and inflammation in pregnancies with gestational diabetes mellitus

In pregnancies affected by gestational diabetes mellitus (GDM), inflammation and adipokines play crucial roles in the pathophysiology of the illness. Recent research has shed light on the intricate pathways through which these factors impact the development of GDM. One of the primary contributors to the development of GDM is inflammation, which not only leads to insulin resistance but also hampers glucose metabolism (137). Women diagnosed with GDM often exhibit elevated levels of pro-inflammatory cytokines such as C-reactive protein (CRP), interleukin-6 (IL-6), and tumor necrosis factor-alpha (TNF-α) (138). These inflammatory mediators disrupt insulin signaling pathways, resulting in insulin resistance and hyperglycemia. Moreover, inflammation exacerbates oxidative stress and endothelial dysfunction, posing additional risks to the health of both the mother and the fetus (139).

Adipose tissue secretes bioactive chemicals known as adipokines, which play a crucial role in regulating inflammation and metabolic balance in GDM. The anti-inflammatory adipokine adiponectin, for instance, enhances glucose absorption and fatty acid oxidation, thereby offering protection against GDM and exhibiting insulin-sensitizing properties (140). Conversely, high levels of pro-inflammatory adipokines like resistin and leptin in women with GDM are associated with insulin resistance and adverse pregnancy outcomes (141). These fat-soluble hormones influence immune responses, trigger inflammatory processes, and contribute to the disruption of glucose metabolism during pregnancy. Numerous research studies have delved into the intricate relationship between adipokines, inflammation, and the pathophysiology of GDM (142, 143). A study conducted by Lain et al. revealed a significant correlation between the onset of GDM and elevated maternal levels of TNF-α and IL-6 (144). Furthermore, research by Catalano et al. showcased the disruption of adipokine profiles, indicating that individuals with GDM exhibited heightened levels of leptin and diminished levels of adiponectin compared to healthy pregnant women (145).

These findings emphasize the importance of understanding the role of adipokines and inflammation in GDM to identify potential treatment targets and improve pregnancy outcomes. Adipokines and inflammation play pivotal roles in the pathophysiology of pregnancies complicated by GDM. Elevated levels of pro-inflammatory cytokines and imbalanced adipokine profiles are associated with insulin resistance, impaired glucose metabolism, and adverse consequences for both the mother and the fetus. To develop innovative therapeutic strategies aimed at mitigating the inflammatory response and restoring metabolic equilibrium in women with GDM, further research is imperative to elucidate the underlying mechanisms.

## 13 Clinical implications and applications of GDM

The clinical implications of gestational diabetes mellitus (GDM) are significant and demand attention from medical professionals worldwide. It is crucial to develop effective screening techniques for early detection and treatment. GDM increases the risk of complications for maternal health, including pre-eclampsia and cesarean sections, emphasizing the need for close observation during pregnancy (146, 147). Furthermore, the long-term consequences for mothers, such as an elevated risk of type 2 diabetes, underscore the importance of postpartum care. GDM is also associated with macrosomia and neonatal hypoglycemia in the fetus, necessitating careful glycemic management to mitigate these risks.

Recent epidemiological studies have indicated that the offspring of women with GDM may have a heightened susceptibility to adverse cardiometabolic outcomes later in life (148). Notably, a comprehensive Danish population-based cohort study (n = 2,432,000) has revealed a significant association between maternal diabetes and an increased incidence of early-onset cardiovascular disease (CVD) in offspring, particularly those aged ≤40 years (149). To shed light on the underlying mechanisms (150), has proposed a potential link between insulin resistance and the activation of inflammatory pathways. Furthermore, *in vitro* research has demonstrated that elevated glucose concentrations impede trophoblast invasiveness by inhibiting uterine plasminogen activator activity (151).

**Figure 3:**
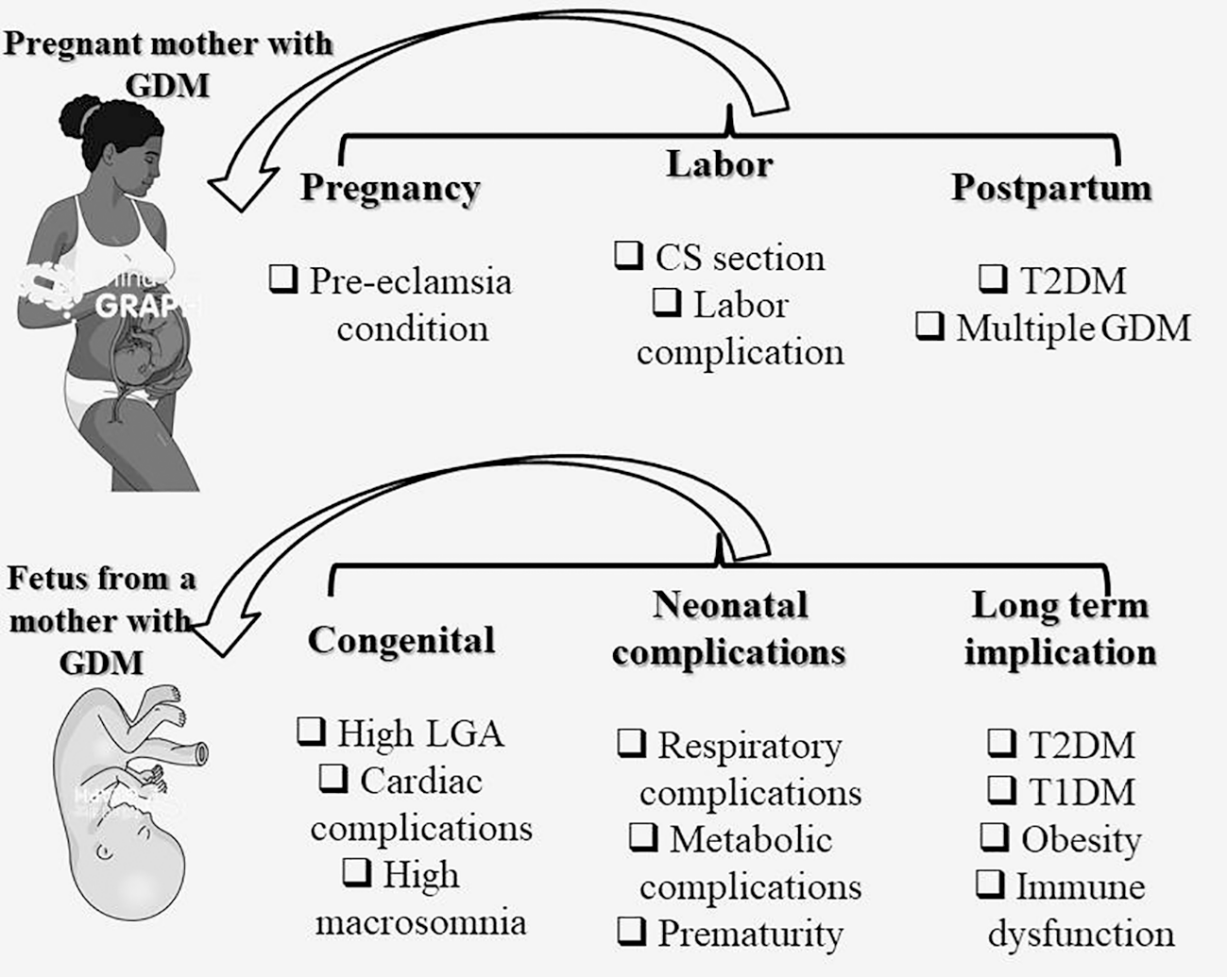
Impact of GDM on the mother and offspring. CS, C-Section (Cesarean delivery); GDM, gestational diabetes mellitus; T2DM, Type 2 diabetes mellitus; T1DM, Type 1 diabetes mellitus; LGA, Large for gestational age.

In light of the significant impact of type 2 diabetes and cardiovascular disease on the worldwide prevalence of intergenerational cardiometabolic disease, it is of utmost importance to acknowledge GDM as an early risk factor for these conditions. Moreover, it is crucial to expand the current clinical approach to encompass long-term complications for both mothers and their offspring following a diagnosis of GDM (152). The management of GDM can be further enhanced by integrating technological advancements, such as telehealth services and continuous glucose monitoring, into clinical practice. These innovations enable real-time sugar level monitoring, facilitating prompt treatment plan adjustments. Given the global epidemic of diabetes, obesity, and cardiovascular disease, the conventional focus on achieving normal obstetric and neonatal outcomes through short-term antenatal maternal glucose management should now shift towards early postnatal prevention strategies. This shift aims to reduce the progression from GDM to type 2 diabetes and address the long-term metabolic risks faced by both mothers and their offspring (152). **Figure 3** depicts the diagram illustrating the impact of GDM on both mothers and their offspring.

## 14 Implications of gestational diabetes mellitus on the influence of cardiovascular disorders

Pregnancy-related Gestational Diabetes Mellitus (GDM) poses a significant health risk, impacting both the short- and long-term health of both mothers and their children. According to the American Heart Association, GDM increases the likelihood of developing cardiovascular disease (CVD) in women (153–155). Research consistently links GDM to future hypertension, dyslipidemia (156, 157), vascular dysfunction, and atherosclerosis (158–160), and other cardiometabolic risk markers (161).

Recent studies, such as by (154, 162), have shed light on the potential impact of GDM on cardiovascular disorders post-childbirth. Research suggests that women with a history of GDM may have an increased susceptibility to CVD, indicating a possible lifelong continuum of metabolic abnormalities (160, 163). For instance, a study conducted by (164) on a large cohort of US women revealed a strong association between a history of GDM and a heightened long-term risk of cardiovascular disease. Additionally (165), found that mothers with GDM had higher rates of pediatric obesity and maternal glucose metabolism abnormalities, underscoring the intricate relationship between pregnancy-related metabolic disruptions and cardiovascular health outcomes. These findings highlight the importance of comprehensive screening, monitoring, and care plans for women affected by GDM to mitigate their long-term risk of cardiovascular disease.

GDM influences CVD through a mechanism of action that may stem from a direct correlation between experiencing a GD pregnancy and cardiometabolic function. Previous prospective and cross-sectional studies have shown that women with a history of GD are more likely to develop atherosclerosis, experience decreased vascular function, and have a higher likelihood of developing dyslipidemia and hypertension compared to women without gestational diabetes (160, 161, 166, 167). Variations in these intermediate phenotypes are observed before the onset of type 2 diabetes, and in some studies, they become apparent shortly after the index pregnancy. It is also suggested that the cardiovascular effects of GD may persist even if type 2 diabetes does not develop, which is a known risk factor for CVD (168).

While few studies have thoroughly examined the prospective link between GDM and eventual CVD while controlling for common risk factors and lifestyle features, a study by (164) found that women with healthier behavior profiles did not have a higher risk of CVD when they had GDM. This association could potentially be explained by controlling for updated lifestyle risk factors for CVD, such as diet, physical activity, smoking status, and weight management. To further investigate whether GDM induces adverse cardiovascular changes, acts as a marker for underlying high risk, or a combination of both, prospective studies with carefully phenotyped CVD markers before and after pregnancy are needed. These studies will help clarify the relationship between GDM and CVD and potentially aid in lowering the risk of developing cardiovascular disease in women with a history of gestational diabetes.

## 15 Conclusion and recommendations

This narrative review explores the involvement of several genes, including SLC30A8, CDKAL1, TCF7L2, IRS1, and GCK, in GDM, along with the adverse effects of stress on insulin function. These factors collectively contribute to the onset of diabetes and the subsequent development of GDM, impacting both the mother and offspring negatively throughout their lives. These genetic variants notably influence beta-cell function and regulate insulin activity in various tissues, disrupting glucose regulation during pregnancy. It is suggested that these variants may disrupt zinc transport, leading to impaired insulin synthesis and secretion, thus contributing to GDM development. Additionally, inflammatory pathways like TNF-alpha and IL-6 can influence susceptibility to GDM by affecting cytokine production and regulation genes. Inflammatory factors such as TNF-α trigger insulin resistance (IR), further complicating glucose metabolism in GDM patients. Furthermore, women with a history of GDM may have an increased susceptibility to CVD, indicating a possible lifelong continuum of metabolic abnormalities. The complex relationship between inflammatory pathways, genetics, CVD and GDM necessitates further research to develop targeted gene therapy and therapeutic drugs aimed at addressing genetic variations in SLC30A8, CDKAL1, TCF7L2, IRS1, GCK, and related genes.

## Author contributions

GR: Conceptualization, Funding acquisition, Resources, Software, Visualization, Writing – original draft, Writing – review & editing. QZ: Conceptualization, Funding acquisition, Supervision, Visualization, Writing – original draft, Writing – review & editing. PK: Software, Validation, Writing – original draft, Writing – review & editing. HZ: Validation, Writing – review & editing. TS: Supervision, Writing – review & editing. TY: Supervision, Writing – review & editing. YW: Supervision, Writing – review & editing. ML: Supervision, Writing – review & editing. XC: Supervision, Writing – review & editing. RG: Conceptualization, Funding acquisition, Resources, Supervision, Validation, Writing – original draft, Writing – review & editing.
